# Extrinsic Trust as a Contractual Framework for Accountable AI in Health Care: Viewpoint

**DOI:** 10.2196/83903

**Published:** 2026-03-05

**Authors:** Anthony Kelly

**Affiliations:** 1 Department of Electronic and Computer Engineering, University of Limerick, Castletroy, Limerick, V94 T9PX, Ireland, 353 61 202700; 2 Health Research Institute, University of Limerick, Limerick, Ireland

**Keywords:** artificial intelligence, AI, explainable artificial intelligence, explainable AI, XAI, trust, machine learning, ML, mental health, decision support system, intrinsic trust

## Abstract

Artificial intelligence (AI) promises efficiency and equity in health care. However, adoption remains fragmented due to weak foundations of trust. This Viewpoint highlights the gap between intrinsic trust, based on interpretability, and extrinsic trust, based on functional validation. We propose a contractual framework between AI systems and users defined by 3 promises: reliability, scope and equity, and shift and uncertainty. Illustrated through a vignette, we show how health systems can operationalize these promises through structured evidence and governance, translating trustworthy AI into accountable clinical deployment.

## Introduction

Artificial intelligence (AI) has the potential to transform clinical care, with applications spanning triage, diagnosis, risk prediction, and resource allocation [1]. However, despite this technical promise, real-world adoption remains fragmented. Trust is a central barrier to adoption as clinicians and decision-makers remain hesitant to rely on AI in safety-critical environments, where errors carry significant consequences for patient outcomes, liability, and public confidence [2].

Adoption requires structured, credible evidence. A recent scoping review identified impediments including limited external validation data, transparency and equity concerns, workflow integration difficulties, and unclear accountability and highlighted facilitators such as robust validation, governance, and postdeployment monitoring [3].

Conceptually, the gap between AI engagement and adoption corresponds to a distinction between intrinsic and extrinsic trust. Intrinsic trust is the sense that clinicians gain when AI outputs are interpretable and aligned with clinical reasoning; extrinsic trust (or functionality trust) [4] rests on empirical evidence that the model performs reliably across diverse populations and under real-world conditions [5]. Intrinsic trust is necessary to encourage engagement and build confidence in the system, but extrinsic trust ultimately influences deployment decisions [5,6].

Establishing extrinsic trust is fundamentally a data and infrastructure challenge. We argue that health systems require structured approaches to generate, verify, and act on validation evidence under real-world conditions. Accordingly, we introduce a contractual framing of extrinsic trust supported by a minimum evidence package and a clinical vignette. Rather than proposing a new reporting artifact, the framework complements existing tools such as model cards, algorithmic audits, and assurance cases by treating trust as a small set of explicit, testable promises. Each promise is linked to defined evidence requirements, verification roles, and breach conditions that trigger governance action across development, deployment, and postdeployment monitoring.

## Intrinsic and Extrinsic Trust in Clinical AI

Clinicians engage with AI systems in stages, beginning with how outputs align with their professional reasoning [7]. This early stage of intrinsic trust develops when predictions are interpretable and presented in ways that resonate with clinical heuristics [8-10].

Intrinsic trust provides a foundation for engagement, but decisions about deployment depend on further evidence. Clinicians require independent validation that the model performs reliably across different patient groups and that it can indicate when deference to human judgment is warranted in the face of predictive uncertainty [11-15]. Interpretable AI models are central to the sensemaking involved in intrinsic trust, but decisions about adoption require evidence of performance under real-world conditions [16]. To express this issue more succinctly, explainability fosters engagement, but validation governs deployment [5,12,14]. Intrinsic trust lowers the barrier to initial use, while extrinsic trust provides the assurance needed for safe integration into workflows and health system decision-making.

Extrinsic trust validates that the model can generalize from the training examples in the dataset to previously unseen examples. As such, it must be either invariant to shift in the statistical distribution of the unseen data or convey increasing uncertainty commensurate with the shift. Since supervised machine learning models are known to be sensitive to out-of-distribution data shift, appropriate validation involves verifying that the model’s sensitivity to out-of-distribution data shift is reliably conveyed through an appropriate uncertainty metric. Shifts within the distribution may occur in subgroups of the data, for example, in demographic or clinical subtypes. Appropriate validation for such in-distribution shifts involves verifying performance invariance. AI model calibration is concerned with how well a model’s predicted probabilities align with actual outcomes and is therefore the foundation of reliability [17]. Calibration provides a foundation for trust by enabling users to gauge whether the AI “knows when it knows.” Without external validation, especially under real-world conditions where data distributions may shift, clinicians may withhold trust or misplace it, leading to underuse or overreliance [12,14,18].

Consequently, the following issues need to be addressed through validation:

- The model should be reliable for in-distribution unseen data examples, demonstrating that predicted probabilities align with actual outcomes.
- Reliability should be maintained for in-distribution subgroups.
- Uncertainty metrics should convey higher uncertainty for out-of-distribution data, demonstrating that the model “knows when it knows.”

Together, these questions define a pragmatic minimum for extrinsic trust rather than a complete taxonomy. They reflect 3 recurrent failure modes in clinical AI deployment: miscalibrated confidence, uneven subgroup performance, and silent degradation under distributional shift. Reliability and uncertainty are complementary. Reliability characterizes confidence alignment within scope, while uncertainty governs safe behavior as that alignment weakens. Scope and equity are treated jointly because subgroup shifts represent clinically salient in-distribution variation that population-level metrics can obscure.

Although these requirements are most relevant for discriminative AI models that predict classifications, there is emerging evidence that generative AI text models (large language models) can be queried about their reliability and uncertainty [19], perhaps allowing for future application of this framework.

## Core Data Requirements for Extrinsic Trust

**Figure 1.**
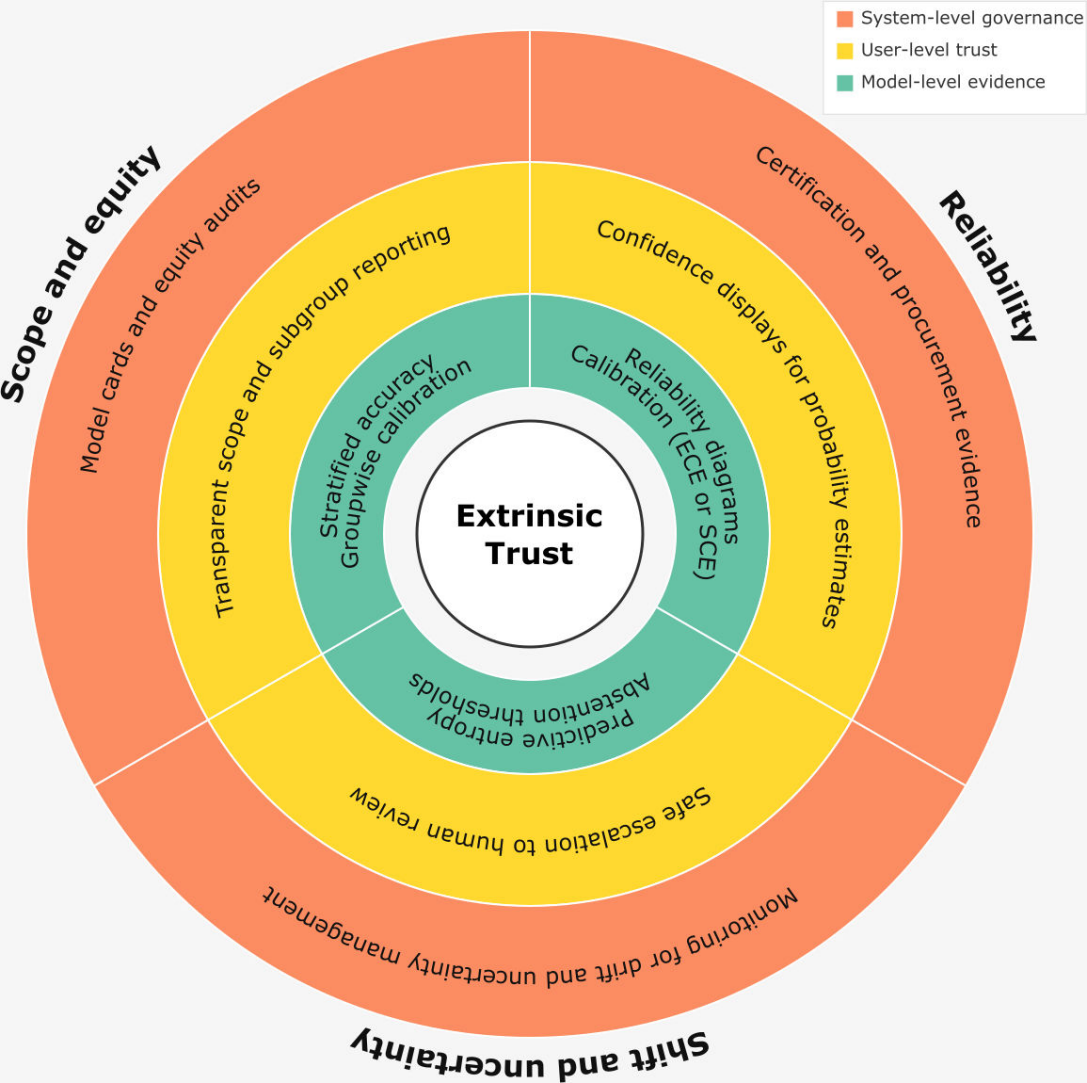
Concentric framework of extrinsic trust. The 3 contractual promises of trust—reliability, scope and equity, and shift and uncertainty—are represented as radial sectors. Each promise operates across 3 levels: model-level evidence (inner ring), user-level trust and workflow fit (middle ring), and system-level governance and accountability (outer ring). At the core lies extrinsic trust, integrating these dimensions into the basis for responsible clinical artificial intelligence adoption. ECE: expected calibration error; SCE: static calibration error.

Similarly to trust in people, trust in AI has been framed as a contract [11,20,21]. Following this notion of contractual trust, users can rely on an AI system when explicit promises are stated and kept. Therefore, users must know what the AI is being trusted with and possess a means of evaluating whether the contract is adhered to [11]. This adherence depends on the context, and the means of determining adherence are dependent on the nature of the contract [8,11]. Drawing on these validation questions, we proposed 3 evidence clauses that should be adhered to for safe deployment (Figure 1) and detail the required metrics in Textbox 1.

Textbox 1. **Metrics for operationalizing the 3 promises of extrinsic trust in artificial intelligence systems.**

**Reliability promise**

- Population-level performance metrics (eg, sensitivity, area under the receiver operating characteristic curve, and *F*_1_-score)
- Expected calibration error or static calibration error: scalar measures of how closely predicted probabilities align with observed outcomes
- Reliability diagrams: visual summaries that clinicians can interpret to judge whether model confidence reflects real-world accuracy

**Scope and equity promise**

- Stratified performance metrics (eg, sensitivity, area under the receiver operating characteristic curve, and *F*_1_-score): reported across clinically and demographically relevant subgroups to detect disparities
- Groupwise calibration plots: showing whether confidence is systematically over or underestimated for particular populations

**Shift and uncertainty promise**

- Negative log-likelihood: evaluates the quality of probabilistic predictions under noise or perturbation
- Predictive entropy or abstention rate: label-agnostic measures of uncertainty, useful for detecting out-of-distribution cases and supporting safe deferral to human review

The term “contractual” refers to a professional and governance compact rather than a legal indemnity framework and does not define liability. It makes explicit the performance promises that an AI system claims to uphold, the evidence required to support them, and the governance actions that follow if they are breached, thereby informing procurement and oversight.

In this compact, developers or vendors make explicit performance claims, health systems commission and govern verification, and clinicians rely on outputs under defined workflow conditions. A promise is meaningful only if keeping or breaching it is observable through agreed artifacts and escalation rules.

Recent work on trust in clinical AI emphasizes that trust is relational and bidirectional, emerging from interactions among models, clinicians, workflows, and institutional safeguards rather than from model properties alone. From this perspective, AI systems depend on human inputs, data quality, and organizational practices to remain safe and effective over time [22]. The contractual promises proposed in this paper are intended to complement this view by specifying the minimum evidence and governance commitments required to sustain trust within such sociotechnical systems, particularly through algorithmic audits and postdeployment monitoring.

## Reliability Promise

The first requirement is a reliability promise: an AI system should provide probability estimates that clinicians can trust in the intended use context. High-confidence predictions should typically be correct, and uncertainty should be visible when the model is unsure.

Reliability depends on calibration: a well-calibrated model produces probability estimates that align with observed outcomes, giving clinicians a clear sense of when the system’s confidence can be relied upon [23,24]. This alignment is crucial in safety-critical settings, where misplaced confidence can lead to harmful decisions. Tools such as reliability diagrams and scalar metrics such as the expected calibration error or its multiclass analogue, the static calibration error (SCE), provide concise ways of demonstrating this alignment by showing a good fit of the reliability curve to the diagonal and low values for the metrics (perfectly calibrated models have an SCE or expected calibration error of 0) [17,25,26]. Monitoring changes in these metrics or the reliability curve fit is useful as acceptable threshold levels are context dependent. Postcalibration scaling can further improve poorly calibrated models [26,27].

For health systems, requiring calibration evidence ensures that AI models offer dependable guidance that supports safe decision-making [12].

## Scope and Equity Promise

The second requirement is a scope and equity promise: AI systems should clearly state who and what they are designed for and then provide evidence that they perform consistently across relevant clinical and demographic subgroups. Declaring scope makes explicit the intended populations, settings, and workflow assumptions, while equity validation ensures that no patient group is systematically disadvantaged.

Validation of the scope and equity promise involves stratified evaluation. Standard performance measures such as accuracy, sensitivity, area under the receiver operating characteristic curve, or *F*_1_-score [28] should be reported not just at the population level but separately for subgroups defined by age, sex, ethnicity, or clinical subtype [29]. Calibration should also be examined within each stratum to detect systematic over- or underestimation. These analyses highlight where models are consistent and where subgroup-specific recalibration or data augmentation may be required. It should be acknowledged that small sample sizes may result from intersectional subgroups that may limit the ability to perform subgroup analysis in some cases.

From a regulatory and governance perspective, AI model cards offer a practical tool for this promise. They function as structured “labels,” detailing intended use cases, target populations, known limitations, subgroup performance, and fairness considerations [30]. This transparency allows clinicians, procurement teams, and regulators to assess whether systems are safe and appropriate for deployment.

Equity is fundamental because health populations are heterogeneous. A model that performs well overall may still underperform in underrepresented groups, amplifying disparities in care [12-14]. Mandating scope declarations and subgroup validation enables health systems to recognize both strengths and limits, supporting targeted safeguards where needed.

## Shift and Uncertainty Promise

The third requirement is a shift and uncertainty promise: AI systems must provide safeguards when confronted with inputs that differ from the data on which they were trained. In evolving clinical environments, populations, referral patterns, and data quality change over time. Without mechanisms to detect and manage such shifts, models risk silent failure in precisely the contexts where reliability is most critical [12,31].

This promise requires evidence that the system can signal uncertainty, degrade gracefully, and defer to human review when appropriate. Metrics such as negative log-likelihood or predictive entropy capture probabilistic quality and uncertainty in unfamiliar cases [26,32,33]. In practice, confidence should decline, and error signals should increase in a predictable manner when inputs deviate or contain errors.

By enabling abstention or escalation under uncertainty, models help protect patients from misleading outputs, preserve equity across heterogeneous populations, and reduce risks of overreliance [34]. Embedding uncertainty clauses into validation frameworks ensures that decision-makers have the information to guarantee that AI remains trustworthy under real-world conditions.

## Minimum Evidence Package for Extrinsic Trust

To formalize the contractual framework, we outline a minimum evidence package (Table 1) that specifies what constitutes proof of each promise, who is responsible for generating and verifying that proof, and what breach conditions require mitigation or suspension of deployment. In many cases, there is no accepted threshold for many of the metrics that constitute proof, although statistical threshold limits may be calculated on held-out calibration sets. For example, a material breach may be defined as a statistically significant upward shift in calibration error relative to the validated deployment baseline as determined by locally agreed monitoring procedures. Therefore, the minimum evidence package provides the foundation for a contract between the AI system and its users: a shared understanding that the model’s behavior is recorded and that deviations can trigger governance actions to mitigate emerging risks before they manifest in clinical practice.

**Table 1.** Minimum evidence package for extrinsic trust. Baselines refer to the model’s validated performance at deployment or the last approved update, with material deviation determined relative to locally defined governance thresholds.

Promise	Artifacts	Producer or verifier	Breach condition and action
All	Model card documenting intended population, features, and workflow assumptions	Producer: vendor or developer Verifier: health system governance and clinical safety officer Trigger: model commissioning and model update	Breach: model card not satisfactory Action: remedial documentation or clarification required before deployment or update
Reliability	Population-level performance metrics (eg, sensitivity, AUC^a^, and *F*_1_-score) Calibration metrics: ECE^b^ or SCE^c^ Reliability diagrams	Producer: vendor or developer Verifier: health system governance and clinical safety officer Trigger: annually; model update or change in patient profiles	Breach: performance or calibration metrics deviate materially from the validated deployment baseline Action: (1) recalibration or retraining, (2) investigation of the root cause, or (3) possible suspension
Scope and equity	Stratified performance metrics (eg, subgroup: sensitivity, AUC, and *F*_1_-score) Groupwise calibration plots	Producer: vendor or developer Verifier: health system governance and clinical safety officer Trigger: annually; model update or change in patient profiles	Breach: subgroup performance or calibration deviates materially from the validated deployment baseline Action: (1) subgroup analysis, (2) recalibration, (3) model card update to exclude subgroups, or (4) possible suspension
Shift and uncertainty	Predictive entropy or abstention rate NLL^d^	Producer: vendor or developer Verifier: health system governance and clinical safety officer Trigger: annually; model update or change in patient profiles	Breach: uncertainty metrics (eg, entropy or NLL) deviate materially from the validated deployment baseline Action: (1) retraining of model with new data, (2) reverification of model, (3) model card update, or (4) possible suspension

a AUC: area under the receiver operating characteristic curve.

b ECE: expected calibration error.

c SCE: static calibration error.

d NLL: negative log-likelihood.

For clinicians and health system decision-makers, the contractual framing also provides a practical cognitive benefit. By organizing technical, regulatory, and statistical requirements into a small number of clear promises, it offers a concise mental model for what trustworthy AI deployment entails, helping cut through the complexity of standards and guidance.

The 3 promises align closely with emerging international regulatory requirements for the analysis and management of AI risks. For example, the European Union AI Act [35] requires transparency (Article 13), accuracy and robustness (Article 15), and risk monitoring (Articles 9 and 72). Although this applies primarily to AI medical devices [36], it nevertheless also implies best practice for lower-risk AI. Table 2 maps each promise to obligations in the European Union AI Act [35], Food and Drug Administration predetermined change control plan [37], and International Organization for Standardization and International Electrotechnical Commission 42001 standard [38], demonstrating that the proposed contractual framework provides a practical implementation framework consistent with regulatory trajectories in high-risk clinical AI.

**Table 2.** Mapping the 3 promises to emerging regulatory requirements.

Promise	EU^a^ AI^b^ Act (Regulation [EU] 2024/1689)	FDA^c^ PCCP^d^	ISO^e^ and IEC^f^ 42001:2023 standard (AI management system)
Reliability promise	Article 15: high-risk AI must achieve appropriate accuracy, robustness, and cybersecurity, and AI providers must declare accuracy metrics and levels and test or validate them Article 9: requires an ongoing risk management system Article 11+annex IV: technical documentation must include information such as performance characteristics and metrics and testing or validation evidence (as specified in annex IV)	The PCCP comprises (1) description of modifications, (2) modification protocol, and (3) impact assessment. The modification protocol should specify verification and validation activities, including performance evaluation methods, performance metrics, statistical tests, and predefined acceptance criteria, and describe how the manufacturer will determine that a modification is acceptable before implementation.	Clause 8.1 (operational planning and control) requires planned and controlled AI operations. Clause 9.1 (monitoring, measurement, analysis, and evaluation) requires organizations to determine what needs to be monitored and measured and how and when to evaluate AI system performance. Clause 10 (improvement) requires correction and continuous improvement when performance deviates.
Scope and equity promise	Article 10: training, validation, and testing data must be sufficiently relevant and representative for the intended purpose, and AI providers must examine and mitigate bias risks Article 13: transparency and instructions enabling appropriate use, including information needed for deployers to interpret outputs and use the system properly	The evidence and testing data supporting the PCCP should be representative of intended use populations (eg, demographic factors where relevant). The PCCP and its modifications must keep the device within the authorized intended use and indications; a change to what was described or authorized (eg, new population not covered) may require a new marketing submission.	Clauses 4.1‐4.3 require definition of organizational context, interested parties, and the scope of the AI management system. Clause 6.1 (actions to address risks and opportunities) requires identification and treatment of AI risks, including risks to individuals and groups. Clause 6.1.4 (AI system impact assessment) requires assessment of impacts on individuals and groups affected by AI systems.
Shift and uncertainty promise	Article 72: AI providers must operate a postmarket monitoring system to collect, document, and analyze performance and risks during real-world use Article 15: robustness expectations (accuracy, robustness, and cybersecurity) support managing performance degradation in foreseeable conditions	The PCCP’s modification protocol should define how the manufacturer will detect the need for change and evaluate modifications; examples include identifying triggers, such as when drift in data is observed. The protocol should specify how performance will be assessed after the change (metrics, tests, or acceptance criteria) and state that modifications should not be implemented if acceptance criteria are not met.	Clause 9.1 requires ongoing monitoring and evaluation of AI systems. Clause 9.2 (internal audit) requires periodic audits to detect nonconformity. Clause 10.2 (nonconformity and corrective action) requires organizations to respond to deviations, investigate causes, and implement corrective actions supporting structured responses to model drift and performance degradation.

a EU: European Union.

b AI: artificial intelligence.

c FDA: Food and Drug Administration.

d PCCP: predetermined change control plan.

e ISO: International Organization for Standardization.

f IEC: International Electrotechnical Commission.

## Case Study Vignette: Operationalizing the 3 Promises Using the Probabilistic Integrated Scoring Model

To illustrate how extrinsic trust can be operationalized, we turn to the Probabilistic Integrated Scoring Model (PrISM) [39], a prototype AI classification system developed for initial treatment screening in routine mental health care. PrISM is a transparent, interpretable neural network that integrates the structured logic of psychometric instruments with probabilistic estimation.

The PrISM study involved a secondary analysis of questionnaire and demographic data from 1068 patients treated in Internetpsykiatrien, Denmark’s national internet-based mental health service. Further details related to this vignette can be found in Multimedia Appendix 1.

**Figure 2.**
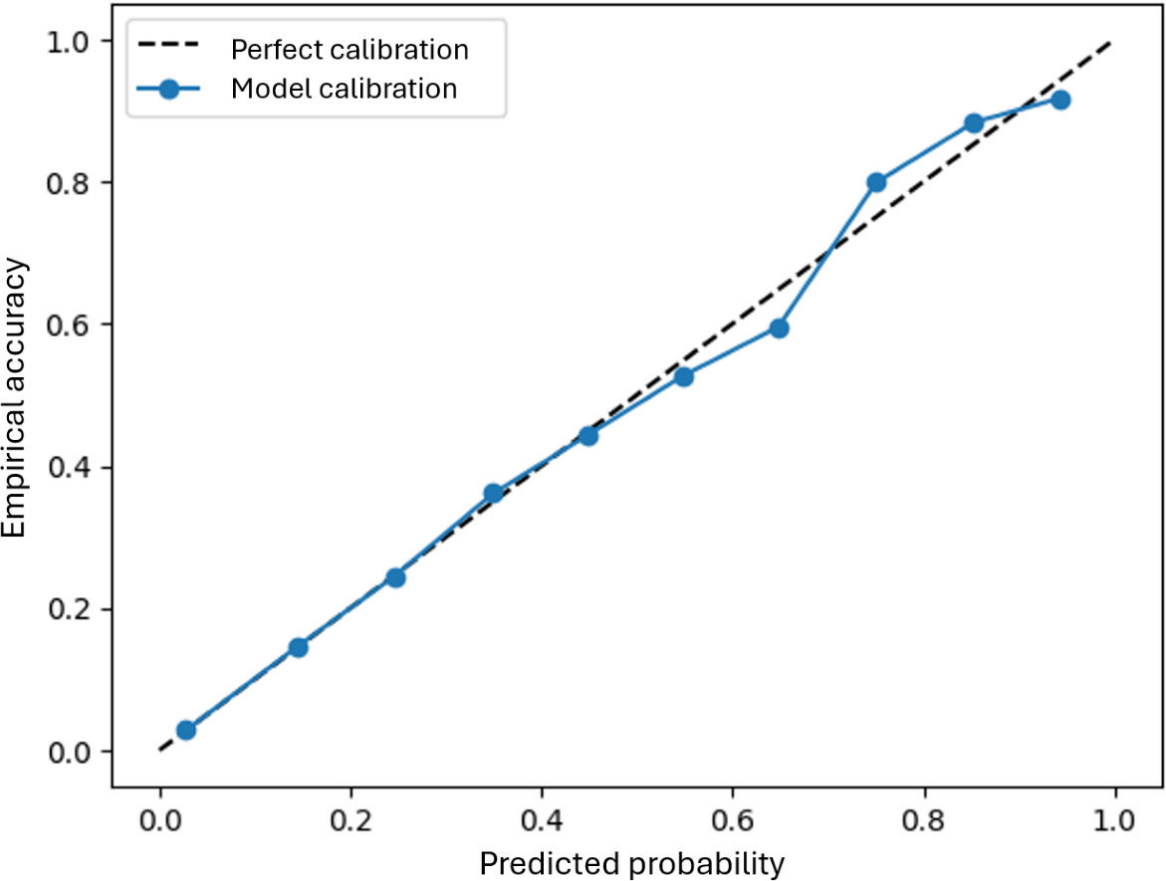
Illustrative example of a reliability diagram for a predictive multiclass model (Probabilistic Integrated Scoring Model). The static calibration error (SCE) of 4.10% and close adherence to the diagonal indicate that the predicted probabilities closely match observed outcomes.

Regarding the reliability promise, reliability diagrams show how closely the model’s predicted probabilities align with observed outcomes. Figure 2 shows that PrISM confidence estimates remain near the ideal diagonal, with an SCE of 4.10%. For a clinician reviewing a referral, this provides assurance that, when the model appears confident, it is generally correct, supporting decisions about when to trust AI guidance and when to seek further input.

Regarding the scope and equity promise, PrISM is explicitly designed and framed as a tool for screening referrals in a digital clinic for mild to moderate depression and anxiety, clearly stating who and what it is designed for. Validation includes stratified calibration and discrimination (eg, by sex or presenting problem), with deployment contingent on subgroup performance remaining within a predefined deviation from the population baseline. This analysis highlights where performance is consistent and where disparities might emerge, enabling clinicians and service managers to anticipate limitations and plan mitigations rather than encountering them unexpectedly in practice.

**Figure 3.**
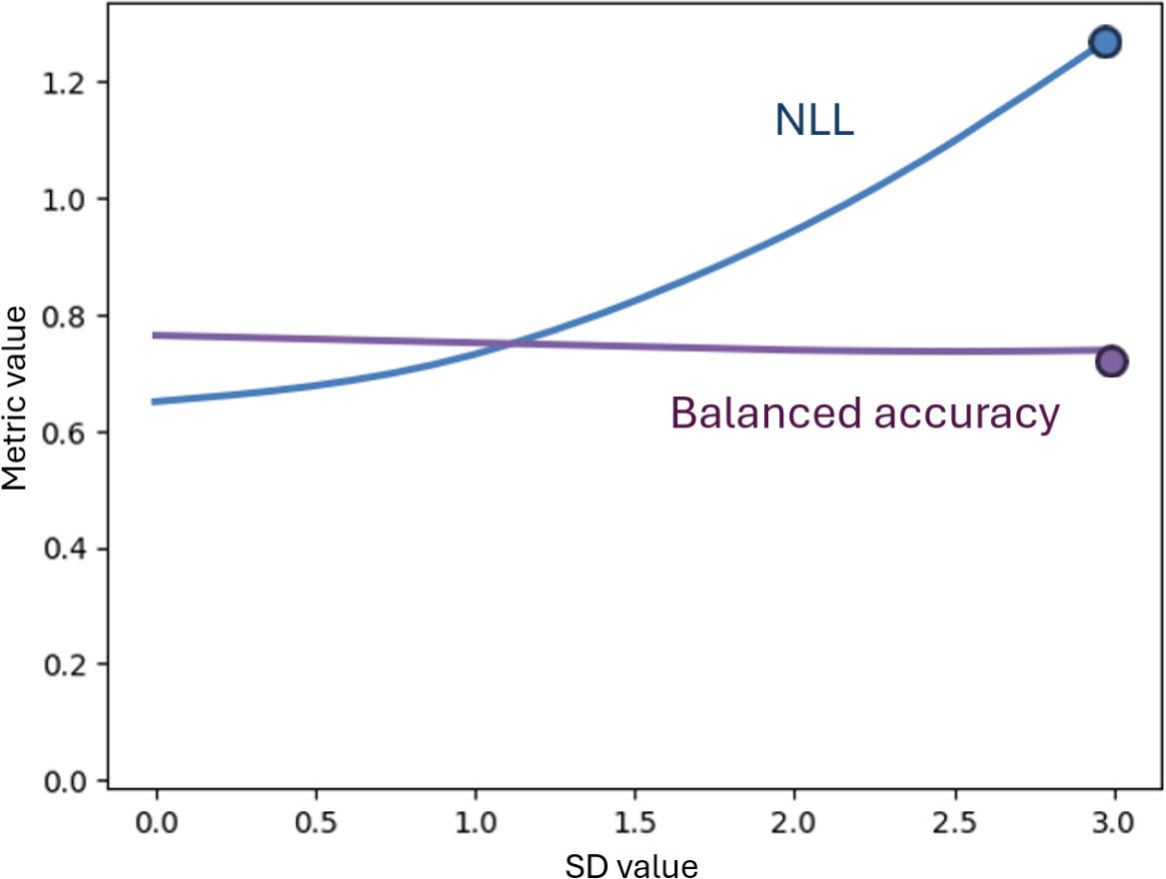
Illustrative example of deterioration of uncertainty (Probabilistic Integrated Scoring Model)*.* As data noise increases (x-axis), the negative log-likelihood (NLL) uncertainty metric increases, whereas the accuracy performance metric stays relatively constant. This illustrates that the model’s performance may be robust to data errors, but the model signals its increasing lack of confidence appropriately.

Regarding the shift and uncertainty promise, in deployment, AI systems encounter cases that differ from their training data. PrISM addresses this by signaling uncertainty. When data inputs contain errors or deviate from their familiar distribution, the uncertainty metric (negative log-likelihood) rises, and the system may abstain from issuing a recommendation, routing the case for human review (Figure 3). For example, cases exceeding the 95th percentile of validation set uncertainty may trigger abstention and clinician review, with abstention rates monitored longitudinally as a safety signal. For clinicians, this safeguard functions as a clear indicator that the model recognizes its limits, ensuring that safe escalation is possible.

This vignette demonstrates that extrinsic trust can be embedded through design choices that make reliability, scope, and uncertainty measurable. The PrISM vignette shows how trust promises can be translated into clinical evidence, allowing health systems to evaluate, monitor, and govern AI as accountable decision support tools.

## Health System Implications and Future Directions

Embedding extrinsic trust requires health systems to translate the 3 contractual promises into routine governance and clinical practice. In practical terms, this means requiring calibration, subgroup performance, and uncertainty evidence at procurement; defining verification roles within clinical safety structures; and specifying escalation actions when promises are breached.

Evidence of trust must be accessible and actionable at the point of care. Simple signals such as confidence or abstention indicators can reduce cognitive burden for frontline clinicians, while dashboards can surface detailed calibration, equity, and drift metrics for governance teams. When systems defer under uncertainty, clear escalation pathways are needed to avoid automation bias or clinician deskilling and preserve human oversight.

Looking ahead, this contractual framing aligns with regulatory shifts toward continuous assurance rather than one-time approval. By treating reliability, scope and equity, and shift and uncertainty as enforceable commitments, health systems gain a lightweight but robust mechanism to govern clinical AI across its life cycle as real-world conditions evolve.

## Conclusions

Extrinsic trust is the decisive factor that determines whether clinical AI remains experimental or becomes a sustainable component of care. While intrinsic trust, supported by interpretability, enables clinician engagement, adoption in safety-critical settings depends on structured evidence of performance under real-world conditions.

By framing extrinsic trust as a contractual relationship defined by 3 promises of reliability, scope and equity, and shift and uncertainty, this Viewpoint provides a practical lens for evaluating, governing, and sustaining clinical AI systems. The PrISM vignette demonstrates that these promises can be operationalized through calibration, subgroup validation, and uncertainty-aware escalation. Together, this approach shifts trust from an abstract aspiration to a testable, accountable basis for responsible clinical AI deployment.

## Supplementary material

10.2196/83903 Multimedia Appendix 1 Details of the Probabilistic Integrated Scoring Model vignette.

## Abbreviations

AI: artificial intelligence
PrISM: Probabilistic Integrated Scoring Model
SCE: static calibration error
