# Supplementary material for: Extrinsic Trust as a Contractual Framework for Accountable AI in Health Care: Viewpoint
Source: J Med Internet Res. 2026 Mar 5;28:e83903. doi: 10.2196/83903 (PMC12978903; doi:10.2196/83903)
Supplement: Multimedia Appendix 1 [file jmir-v28-e83903-s001.DOCX]

### PRiSM Data and Ethics

The PrISM study involved a secondary analysis of questionnaire and demographic data from 1,068 patients treated in Internetpsykiatrien, Denmark’s national internet-based mental health service, between November 14, 2019, and December 31, 2022. After preprocessing, 91 features were retained, and the dataset was split into training (n = 801) and test (n = 267) sets for accuracy evaluation (unless otherwise stated). Data extraction was approved by the Regional Council in Southern Denmark, and the Regional Committees on Health Research Ethics were notified (case S-20232000-65); under national guidelines, no further ethics approval or informed consent was required. The study was reported to the Danish Data Protection Agency, and all data were anonymized prior to researcher access.

### PRiSM Reliability

A well-calibrated model assigns higher confidence to correct predictions and lower confidence to incorrect ones. Expected calibration error (ECE) is a popular scalar summary metric of model calibration that summarizes the average difference between a model’s predicted confidence and actual correctness. ECE partitions predictions into bins based on confidence levels and compares the predicted versus actual accuracy within each bin. An ECE of zero would mean perfect calibration. ECE quantifies the discrepancy between predicted confidence of the top-1 probability vector class and observed accuracy on a dataset across a range of prediction confidence levels, so an ECE of zero corresponds to perfect calibration of the model on the dataset. ECE is defined as:

$$ECE=\sum_{b=1}^{B} \frac{n_{b}}{N}\left| acc\left( b \right)-conf(b) \right|$$

ECE is a sum over B equidistant bins which group the data according to the confidence (prediction probability) assigned to each data point. In the equation, acc(b) represents the true fraction of positive instances in bin b, conf(b) the mean of the probabilities (confidence) for the instances in bin b, n_b_ is the number of predictions in bin b and N the total number of datapoints.

However, ECE, with its origins in binary classification, is computed only over the top-1 predicted class for each instance, limiting its applicability in multiclass settings where the full distribution over classes is relevant. Static calibration error (SCE) [28] evaluates each of the output classes independently, computing an average per-class ECE. SCE is defined as:

$$SCE=\frac{1}{K}\sum_{k=1}^{K} \sum_{b=1}^{B} \frac{n_{bk}}{N}\left| acc\left( b,k \right)-conf(b,k) \right|$$

Here, acc(b,k) is the true fraction of positive instances in bin b and class k. Similarly, conf(b,k) is the mean of the probabilities (confidence) for the instances in bin b and class k; n_bk_ represents the number of predictions in class k of bin b. Divided by N (the total number of data points) this represents the fraction of all instances of class k that fall into bin b. K is the number of classes and B is the number of bins. The inner sum of the SCE equation is effectively a per-class ECE, computed by treating the predicted probability for each class as a separate binary classifier. By treating each class as a separate binary classification problem, SCE captures calibration across the full predictive distribution in multiclass classification tasks.

In this vignette the SCE is calculated on 10 equal bins of 10% range from 0% to 100%.

To complement scalar calibration metrics such as SCE, we use reliability diagrams to provide a visual representation of calibration performance. In the multiclass setting, these diagrams plot predicted confidence (binned) against observed accuracy, averaged over all classes. The SCE reliability diagram provides a global summary of how well predicted probabilities align with actual outcomes across the full predictive distribution. A perfectly calibrated model will produce a curve that lies along the diagonal, whereas deviations from this line indicate over- or under-confidence in the model’s probability estimates.

### PRiSM Uncertainty

Moving beyond calibration, predictive uncertainty metrics help clinicians know when the model is unsure, whether due to ambiguous inputs or unfamiliar patient profiles (for example). While calibration is concerned with the correspondence between confidence and observed frequencies, predictive uncertainty metrics assess how certain or uncertain a model is about its predictions. One widely used measure of predictive uncertainty is the Negative Log Likelihood (NLL), which quantifies the quality of probabilistic predictions. The Negative Log Likelihood (NLL) for multiclass classification is defined as:

$$NLL=-\frac{1}{N}\sum_{i=1}^{N} \log\left( p_{i,y_{i}} \right),$$

where p_i,yi_ is the predicted probability of the true class y_i_ and y_i_ is the index of the true class label for instance i and the sum ranges across N datapoints. The NLL penalizes incorrect and overconfident predictions by assigning a high loss when the predicted probability for the true class is low. A lower NLL indicates that the model assigns high probability to the correct classes, thus better capturing the underlying data distribution.

### PRiSM Entropy

Unseen data examples may arise in new clinical settings or as a result of unexpected variations in patient presentation, where established ground truth labels are either unavailable or inapplicable. In such out-of-distribution (OOD) scenarios, traditional evaluation metrics—including Negative Log Likelihood (NLL), and Static Calibration Error (SCE), are not suitable. These metrics depend on comparing predicted probabilities to known labels, and thus cannot be applied when evaluating model performance on label-free test sets. As a result, alternative approaches are needed to assess model reliability and uncertainty in these settings.

Predictive entropy serves as a label-agnostic measure of model uncertainty. It captures the dispersion of the model’s output probability distribution, allowing the evaluation of how confident the model is about its predictions without needing any reference to correctness.

For a single input with predicted class probabilities p=[p1,p2,…,pK]p=[p1​,p2​,…,pK​], the predictive entropy is defined as:


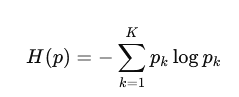


where K is the number of output classes. Entropy reaches its minimum (0) when the model is fully confident in one class (i.e., pk=1 for some k), and is maximized when the model is completely uncertain (i.e., pk=1/K​ for all k).

To summarize uncertainty across an entire dataset of N inputs, the mean predictive entropy is calculated as:


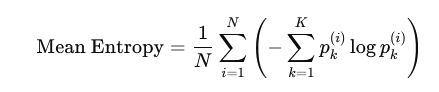


This provides a scalar estimate of the model’s average uncertainty over the dataset. In the context of diagnostic OOD evaluation, where the test set may include inputs from previously unseen conditions (e.g., PTSD, OCD, or adjustment disorder in a model trained only on depression, panic, social phobia, and specific phobia), we expect a well-behaved model to yield higher mean entropy—indicating that it is appropriately uncertain about unfamiliar cases.

Entropy-based approaches are widely used in threshold-based OOD detection, where predictions exceeding a specified entropy threshold are flagged as uncertain or rejected altogether. While entropy alone may not distinguish all forms of OOD reliably—particularly when models are poorly calibrated or overconfident—it remains one of the few applicable and interpretable tools in label-free OOD verification. Its effectiveness can be further enhanced when used alongside techniques such as temperature scaling, ensemble modeling, or uncertainty-aware training.

### PRiSM Calibration

To assess model calibration (a measure of reliability), we employed a Monte Carlo Dropout-based K-Fold cross-validation procedure (K=5). To maximise the use of the data available for the calibration, the PrISM model was trained using stratified K-fold splits of the entire dataset (n=1,068), with out-of-fold predictions aggregated across folds to ensure each evaluation sample was unseen during training. At inference, Monte Carlo Dropout was used to generate multiple stochastic forward passes, capturing epistemic uncertainty. Predicted probabilities were averaged across passes to improve calibration reliability. Calibration was evaluated using per-class ECE, SCE, and the Brier score, computed from the aggregated predictions. To reduce variance from fold selection, and model training, the entire procedure was repeated four times and results were smoothed across runs.

The model demonstrated strong calibration performance, with a Static Calibration Error (SCE) of 4.1% indicating that predicted probabilities closely matched observed outcomes. This is further supported by the SCE reliability diagram, where the calibration curve follows the ideal diagonal line across most probability bins, with only minor deviations in the mid-range (0.35–0.45), suggesting slight underconfidence. Overall, the model’s predicted probabilities appear both reliable and interpretable, supporting its potential use in downstream clinical decision-making contexts.

*PRiSM Shift and Uncertainty promise: Synthetic data perturbation (noise)*

In another set of experiments, we tested the response of the model to additive Gaussian noise (AGN) perturbations of its internal scoring mechanism and demographic features. Specifically, we introduced AGN to 31 inputs of the softmax output layer of the PrISM model. The AGN standard deviation was varied between 0 and 2, with a mean of 0. This approach simulates gradual degradation in input quality or measurement reliability, such as might occur due to inconsistent questionnaire responses, missing values imputed with noise, or data entry errors in demographic fields. By incrementally increasing the standard deviation of the noise, we assessed the robustness of the model’s confidence estimates across a continuum of signal-to-noise conditions.

Figure 4 in the main paper text, illustrates the effect of Gaussian noise on various uncertainty and performance metrics. The noise is injected into the softmax logits, representing the total scores of PHQ (Patient Health Questionnaire score), GAD (Generalized Anxiety Disorder score), SIAS (Social Interaction Anxiety Scale score) and PDSS (Panic Disorder Severity Scale score). As the standard deviation of the noise increases, Negative Log Likelihood (NLL), Brier score, and Scaled Calibration Error (SCE) all increase, reflecting a decline in prediction confidence. However, balanced accuracy remains largely unchanged, suggesting that the noise perturbs the model's confidence without substantially affecting its top-1 predictions.

This behaviour suggests that the model is capable of expressing meaningful uncertainty: as artificial noise is introduced, the confidence-based metrics (e.g., NLL, Brier score, SCE) deteriorate while accuracy remains stable. This decoupling implies that the model's uncertainty estimates are responsive to noise even when its class predictions are unaffected — a desirable property in high-stakes or safety-critical applications where knowing when not to trust a prediction is as important as the prediction itself.
